# In Vitro–In Silico Modeling of Caffeine and Diclofenac Permeation in Static and Fluidic Systems with a 16HBE Lung Cell Barrier

**DOI:** 10.3390/ph15020250

**Published:** 2022-02-18

**Authors:** Lukas Kovar, Lena Wien, Dominik Selzer, Yvonne Kohl, Robert Bals, Thorsten Lehr

**Affiliations:** 1 Department of Clinical Pharmacy, Saarland University, 66123 Saarbrucken, Germany; lukas.kovar@uni-saarland.de (L.K.); dominik.selzer@uni-saarland.de (D.S.); 2 Fraunhofer Institute for Biomedical Engineering IBMT, 66280 Sulzbach, Germany; lena.wien@ibmt.fraunhofer.de (L.W.); yvonne.kohl@ibmt.fraunhofer.de (Y.K.); 3 Department of Internal Medicine V, Saarland University, 66421 Homburg, Germany; robert.bals@uks.eu

**Keywords:** in vitro–in silico modeling, 16HBE, caffeine, diclofenac, fluidic system, static system

## Abstract

Static in vitro permeation experiments are commonly used to gain insights into the permeation properties of drug substances but exhibit limitations due to missing physiologic cell stimuli. Thus, fluidic systems integrating stimuli, such as physicochemical fluxes, have been developed. However, as fluidic in vitro studies display higher complexity compared to static systems, analysis of experimental readouts is challenging. Here, the integration of in silico tools holds the potential to evaluate fluidic experiments and to investigate specific simulation scenarios. This study aimed to develop in silico models that describe and predict the permeation and disposition of two model substances in a static and fluidic in vitro system. For this, in vitro permeation studies with a 16HBE cellular barrier under both static and fluidic conditions were performed over 72 h. In silico models were implemented and employed to describe and predict concentration–time profiles of caffeine and diclofenac in various experimental setups. For both substances, in silico modeling identified reduced apparent permeabilities in the fluidic compared to the static cellular setting. The developed in vitro–in silico modeling framework can be expanded further, integrating additional cell tissues in the fluidic system, and can be employed in future studies to model pharmacokinetic and pharmacodynamic drug behavior.

## 1. Introduction

With the introduction of the European Directive 2010/63/EU on the protection of animals used for scientific purposes [1], the 3R principles, which aim to replace, reduce, and refine preclinical animal studies [2], received legal recognition for the first time. For this, further investigations into how in vitro and in silico approaches can support overcoming the limitations of current preclinical methods whilst reducing the need for animal studies are required [3].

In vitro permeation experiments can be used to characterize the transport of drug molecules across biological barriers, such as the pulmonary [4,5,6] or intestinal epithelia [7,8,9] barrier. The information gained in permeation studies on the properties of drug substances or environmental pollutants and their interaction with permeation barriers can be employed to estimate transport through physiologic endothelial and epithelial barriers in vivo [7,10,11,12]. A common approach for investigating the respective apparent permeability through a cell monolayer in vitro is the application of a single-barrier model [9,11,13]. Here, the apparent permeability is a composite parameter that not only comprises diffusion through the apical and basolateral membrane but that also considers additional processes such as diffusion through unstirred aqueous layers or through porous filter membranes and that has been successfully used for the prediction of in vivo absorption processes [7,10,11].

However, while static in vitro cell culture experiments are still widely used to investigate research questions, static monoculture approaches show several limitations regarding missing cell stimuli that are present in vivo [14,15]. One major group of missing stimuli is that of physicochemical fluxes [14,15]. To integrate this stimulus and to generate more predictive in vitro models, the development and application of fluidic and microfluidic systems have been proposed as early stage approaches for the investigation of drug compounds and complex biological processes [14,16,17,18,19,20].

Fluidic systems show distinct advantages over their static counterparts, as they hold the potential to mimic physiologic conditions more closely, replicating the physiological flows of tissue fluids, introducing shear stress, enabling the spatiotemporal control of medium supply, and ensuring waste removal, among other aspects [17,21,22]. Additionally, by integrating multiple tissues in multi-organ fluidic systems, tissue interactions and physiological processes, such as the bioactivation of compounds, can be simulated [21,22,23,24,25]. However, the interpretation and translation of the corresponding in vitro readouts are challenging [19].

Here, in silico modeling techniques that are already widely applied in drug discovery and development [26,27,28] can be helpful tools to support study design, data analysis, and extrapolation of in vitro studies [19,29,30]. The integration of in vitro and in silico modeling approaches offers the opportunity to quantitatively evaluate the results of fluidic in vitro systems and to use simulation techniques to predict yet untested scenarios [7,29,31]. Moreover, in silico models hold the potential to integrate prior knowledge into simulation experiments to help inform decisions related to study design (e.g., time of sampling) and to gain information on a drug’s pharmacokinetic and pharmacodynamic behavior, allowing extrapolation of drug doses and decreasing the need for in vivo studies [7,25,29,31].

In this study, the modular MultiCompartmental Bioreactor (MCmB) array Quasi-Vivo^®^ was employed [16,20,32,33,34], and an in silico model mimicking the in vitro fluidic experimental system was implemented. The in silico model was used to simulate concentration–time profiles of the investigated probe substances caffeine and diclofenac. For model predictions in the fluidic system, the apparent permeability of the substances was estimated based on static in vitro experiments that were conducted to obtain prior knowledge regarding the experimental setup and the model substances.

Thus, the presented work aimed to develop in silico models to describe and predict the permeation and disposition of the two model substances, caffeine and diclofenac, in a static and fluidic in vitro system. For this, a compartmental modeling approach was employed to simulate drug concentration–time profiles over a period of 72 h after single-dose administration. The in vitro experiments were performed both for a control scenario (transfer through a porous membrane without cells) and for permeation through a 16HBE epithelial cell monolayer cultured on a porous membrane. Subsequently, the experimental setups were assessed with the implemented in silico models.

## 2. Results

In vitro permeation studies with 16HBE cells (cell-based experiments) and without cells (control) were performed under static and fluidic conditions. At eight different time points (15 min–72 h), the apical and basolateral concentrations of the two probe substances (caffeine and diclofenac) were quantified via high-performance liquid chromatography (HPLC). Based on these data, in silico models mimicking the in vitro setup were developed to stimulate concentration–time profiles of the model substances.

Before performing the permeation studies, the 16HBE cell model was established to control the integrity of the cell layer and the viability of the cells. The integrity of the 16HBE cell layer was assessed using transepithelial electrical resistance (TEER) measurements, showing TEER values of 671.46 Ω*cm^2^ ± 46.21 Ω*cm^2^. The viability of the lung cells was verified by an alamarBlue™ assay. Compared to the cell viability in the static system, which was defined as 100% with a standard deviation of 15.2% (*n* = 3), the viability under fluidic conditions was 118.9% ± 7.1% (*n* = 3), while the difference was not statistically significant (*p* < 0.05).

### 2.1. Static Permeation Studies

**Figure 1:**
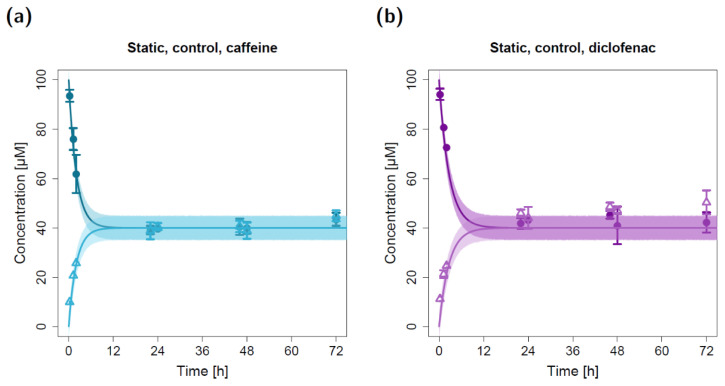
Apical and basolateral concentration–time profiles for caffeine (**a**) and diclofenac (**b**) in the static permeation study without lung cells (control). Darker colors show apical concentrations, while lighter colors show basolateral concentrations, both observed and simulated. Observed concentrations are depicted as solid circles (apical) and open triangles (basolateral) (arithmetic mean and standard deviation, *n* = 5 for caffeine, *n* = 3 for diclofenac). Model simulations are shown as lines, and shaded areas depict the 68% simulation prediction interval.

**Figure 2:**
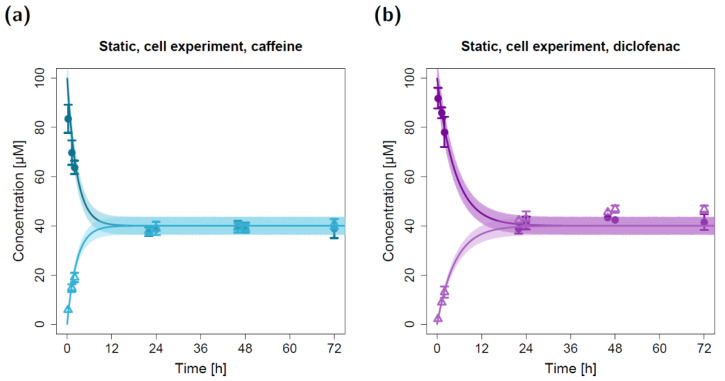
Apical and basolateral concentration–time profiles for caffeine (**a**) and diclofenac (**b**) in the static cell permeation study. Darker colors show apical concentrations, while lighter colors show basolateral concentrations, both observed and simulated. Observed concentrations are depicted as solid circles (apical) and open triangles (basolateral) (arithmetic mean and standard deviation, *n* = 5 for caffeine, *n* = 3 for diclofenac). Model simulations are shown as lines, and shaded areas depict the 68% simulation prediction interval.

For the in silico modeling of caffeine and diclofenac permeation in fluidic systems, in vitro studies under static cultivation conditions were performed to obtain prior information on model substance behavior in the experimental setup. In the static permeation experiments, the apical concentrations of both caffeine and diclofenac substantially decreased within the first two hours (first three measurements). Concurrently, the basolateral concentrations increased and reached equilibrium with the apical concentrations after ~22 h (Figure 1 and Figure 2).

For the static permeation experiments without lung cells (control), model parameter estimation resulted in apparent permeabilities (P_12_) of 3.27 × 10^−3^ cm/min for caffeine and 2.51× 10^−3^ cm/min for diclofenac (Table 1). With these input parameters, the observed rapid decline in apical concentrations and the observed rapid incline in basolateral concentrations were well described by the static in silico model (Figure 1). Since no cellular barrier was involved in the static control experiments, an equilibrium was reached between the apical and basolateral compartments that yielded similar concentrations in both chambers at the end of the experiment. Of note, several quantified basolateral diclofenac concentrations (t ≥ 22 h) were slightly elevated compared to the corresponding model-simulated concentrations (Figure 1b). Further, the model simulations suggest that the observed equilibrium might have already been reached after ~10 h for caffeine and after ~14 h for diclofenac (Figure 1).

To model the static in vitro cell permeation studies, the experimental data supported the assumption of equal permeabilities from the apical to the basolateral compartment and vice versa since an equilibrium with similar apical and basolateral concentrations of ~40 µM was reached for both of the modeled compounds after ~24 h (non-polarized permeation, Figure 2). The application of the estimated permeabilities (Table 1) of 2.62 × 10^−3^ cm/min for caffeine and 1.38 × 10^−3^ cm/min for diclofenac led to a close concordance between the experimentally quantified (in vitro) and model-simulated (in silico) apical and basolateral concentrations. Similar to the static control permeation experiment, several observed basolateral diclofenac concentrations (t ≥ 22 h) were slightly elevated compared to the model-simulated concentrations (Figure 2b). Additionally, the model simulations suggest that for caffeine, equilibrium might have already been reached after ~12 h (Figure 2a).

The estimated apparent permeabilities and residual variabilities for the static permeation experiments (both control and cell experiments) are shown in Table 1.

Of note, the proportional residual error term in the in silico model for the control experiments was estimated to be equal to 0 and was hence fixed to this value to ensure proper calculation of the covariance matrix and the resulting residual errors.

### 2.2. Fluidic Permeation Studies

**Figure 3:**
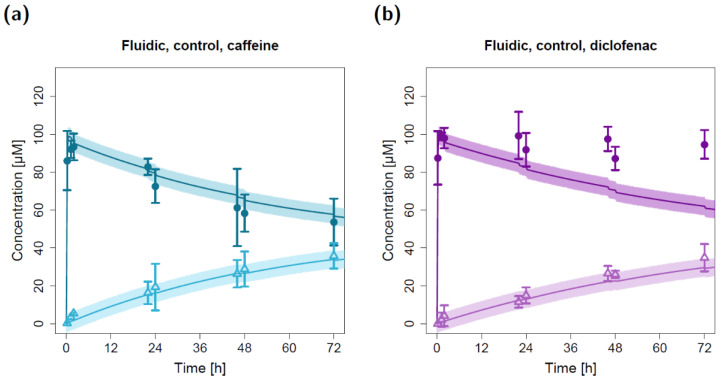
Apical and basolateral concentration–time profiles for caffeine (**a**) and diclofenac (**b**) in the fluidic permeation study without lung cells (control). Darker colors show apical concentrations, while lighter colors show basolateral concentrations, both observed and predicted. Observed concentrations are depicted as solid circles (apical) and open triangles (basolateral) (arithmetic mean and standard deviation, *n* = 3). Model-predicted concentrations represent concentrations in the C_2_ (apical) and C_9_ (basolateral) compartment. Model simulations are shown as lines, and shaded areas depict the 68% simulation prediction interval.

**Figure 4:**
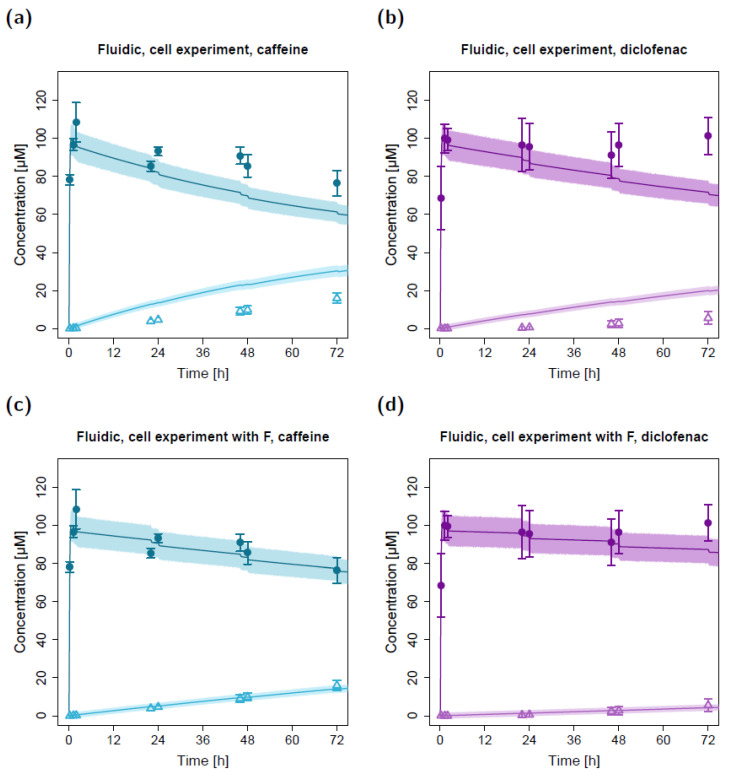
Apical and basolateral concentration–time profiles for caffeine (**a**) and diclofenac (**b**) in the fluidic cell permeation study without (**a**,**b**) and with adjusted permeabilities (**c**,**d**). Darker colors show apical concentrations, while lighter colors show basolateral concentrations, both observed and simulated. Observed concentrations are depicted as solid circles (apical) and open triangles (basolateral) (arithmetic mean and standard deviation, *n* = 4 for caffeine, *n* = 3 for diclofenac). Model-predicted concentrations represent concentrations in the C_2_ (apical) and C_9_ (basolateral) compartments. Model simulations are shown as lines, and shaded areas depict the 68% simulation prediction interval. F, estimated factor to adjust the apparent permeabilities in the fluidic compared to the static setting.

The in silico model for the fluidic system was applied to predict caffeine and diclofenac concentrations in the fluidic system by using the estimated apparent permeabilities derived from the static permeation experiments. All other model input parameters (i.e., compartment volumes and flow rates) were fixed to experimentally determined values. Hence, the depicted model simulations in Figure 3 (control) and Figure 4a,b (cell-based experiments) represent pure predictions of apical and basolateral concentrations based on the static in vitro–in silico modeling platform.

The resulting model simulations for the fluidic study without a lung cell barrier led to precise predictions of the measured caffeine concentrations in both the apical and basolateral circuits (Figure 3a). While the model predictions of the basolateral diclofenac concentrations were in close agreement with the observed data, the measured apical diclofenac concentrations barely decreased during the experiment and were elevated compared to model-simulated concentrations (Figure 3b).

Due to repeated sampling without solvent replacement (eight times) at both the apical and basolateral three-way cock (hereafter referred to as the sampling and dosing port, respectively), the total volume of the apical and basolateral circuits decreased from 15 mL to 11 mL. This resulting reduction in volume (and amount of compound) led to a step-like decrease in simulated apical concentrations at the sampling time points that could also be detected in the observed concentrations (Figure 3).

Using the estimated apparent permeability parameters from the static cell experiments, substance transfer from the apical to the basolateral circuit was overpredicted in the fluidic system for both caffeine and diclofenac (Figure 4a,b). After adapting the apparent permeabilities from the static system (factor (F) of 0.34 for caffeine and 0.17 for diclofenac, respectively), the observed concentration–time courses in the fluidic system could be described well with the in silico model (Figure 4c,d). The resulting permeabilities (P_16_; apparent permeability between the apical and basolateral chambers in the fluidic system) were 8.92 × 10^−4^ cm/min for caffeine and 2.35 × 10^−4^ cm/min for diclofenac.

**Figure 5:**
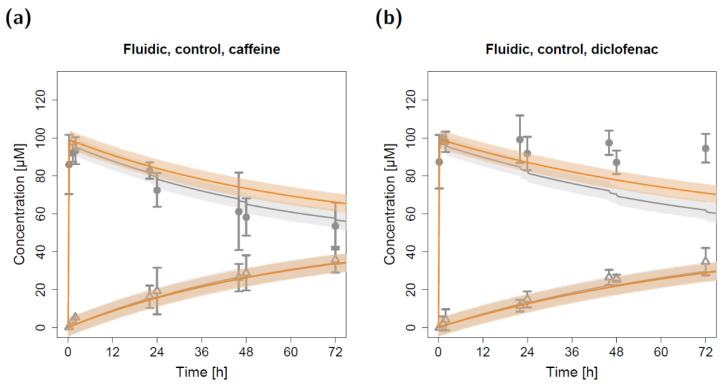
Apical and basolateral concentration–time profiles for caffeine (**a**) and diclofenac (**b**) in the fluidic control permeation study with (grey) and without (orange) considering volume and substance removal. Observed concentrations are depicted as solid circles (apical) and open triangles (basolateral) (arithmetic mean and standard deviation, *n* = 3). Model-predicted concentrations represent concentrations in the C_2_ (apical) and C_9_ (basolateral) compartments. Model simulations are shown as lines, and shaded areas depict the 68% simulation prediction interval.

Model simulations for the fluidic system, which are depicted in Figure 5, show the impact of repeated sampling in the in silico model on apical and basolateral concentrations. While considering the decreasing volume and, consequently, decreasing amount of drug substance led to precise predictions of the measured caffeine in vitro concentrations (Figure 5a, grey profiles), disregarding the sampling would have resulted in an overprediction of the apical concentrations. In contrast, the effect on basolateral concentrations was negligible. Of note, for diclofenac, both implemented approaches led to an underprediction of the apical concentrations (Figure 5b).

**Figure 6:**
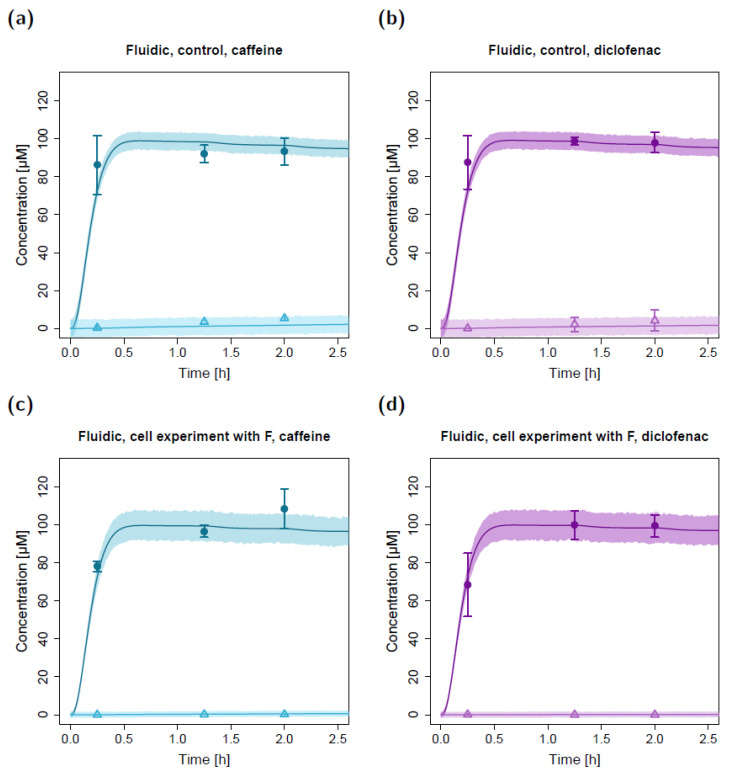
Zoom into the first 2.5 h of apical and basolateral concentration–time profiles for caffeine (**a**,**c**) and diclofenac (**b**,**d**) in the fluidic permeation study without lung cells (control; **a**,**b**) and with lung cells and adjusted permeabilities (**c**,**d**). Darker colors show apical concentrations, while lighter colors show basolateral concentrations, both observed and simulated. Observed concentrations are depicted as solid circles (apical) and open triangles (basolateral) (arithmetic mean and standard deviation, *n* = 3 in (**a**,**b**,**d**) and *n* = 4 in (**c**)). Model-simulated concentrations represent concentrations in the C_2_ (apical) and C_9_ (basolateral) compartments. Model simulations are shown as lines, and shaded areas depict the 68% simulation prediction interval. F, estimated factor to adjust the apparent permeabilities in the fluidic compared to the static setting.

In the fluidic experimental setup, the apical concentrations measured at time t = 15 min were lower compared to the concentrations measured at t = 75 min and t = 120 min (Figure 6). This was in agreement with model simulations where apical concentrations at the sampling port increased during the first ~45 min until equilibrium was reached in the apical circuit (depicted in Figure 6, zooming into the first 2.5 h of the fluidic concentration-time profiles).

## 3. Discussion

The presented work aimed to develop in silico models to describe and predict the permeation and disposition of model substances in a static and fluidic in vitro system. For this, a compartmental modeling approach was used to simulate drug concentration–time profiles over a period of 72 h. In vitro experiments were performed with 16HBE epithelial cells, which were qualified for the permeation studies based on characteristic TEER values [4,35,36] and the non-significant difference in cell viability between static and fluidic cultivation conditions.

Based on the implemented in vitro–in silico modeling framework, concentration–time profiles of the two model substances in both static and fluidic conditions could be described and predicted. Caffeine, a rather hydrophilic (logP~−0.2) and uncharged molecule [37,38], and diclofenac, a highly lipophilic (logP~4) and mainly charged molecule [38,39], were selected as model substances to evaluate the applicability of the in vitro–in silico system.

The experimental data that were collected in the static cellular study suggested that neither caffeine nor diclofenac were subject to active transport regarding the human bronchial epithelial cell line 16HBE since an equilibrium with similar apical and basolateral concentrations was reached at the end of the in vitro experiments. Hence, the permeabilities from the apical to the basolateral compartment and vice versa were assumed to be equal. The bidirectional single-barrier model was suitable to describe the concentration–time profiles in the static system for both the control and the cell-based studies and successfully led to the estimation of apparent permeabilities regarding the transfer of both model substances.

Using the estimated apparent permeabilities from the static in vitro experiments, the fluidic control experiments could be well predicted for the apical and basolateral caffeine concentrations as well as for the basolateral diclofenac concentrations.

In contrast, when employing the estimated apparent permeabilities from the static in vitro study for the fluidic 16HBE cell experiments, caffeine and diclofenac transfer to the basolateral circuit was overpredicted. This could be due to the fact that cellular behavior can differ in fluidic compared to static systems, as shown for cell proliferation, biotransformation, and/or up-regulation of proteins and metabolic enzymes, among others [21,22,40,41,42]. As a result, transfer through the lung cell layer might have been altered in the fluidic compared to the static system due to changes in, for example, protein expression and cell morphology. After empirically adapting the permeabilities from the static in vitro studies, the in silico model could also precisely describe the experimental results from the lung cell barrier study. Here, the estimated adaption factors were 0.34 for caffeine and 0.17 for diclofenac, implying a highly reduced apparent permeability compared to the static in vitro experiments. This case study suggests that estimated apparent permeabilities from static in vitro systems could require refinement or adaptation before application for predictions in fluidic systems.

The estimated apparent permeabilities of 2.62 × 10^−3^ cm/min (static system) and 8.92 × 10^−4^ cm/min (fluidic system) for caffeine in the cell-based experiments are within the same order of magnitude with a published value from a study by Mathias et al. (1.31 × 10^−3^ cm/min) that used the human bronchial epithelial cell line Calu-3 [5]. The apparent permeabilities for diclofenac were estimated to be 1.38 × 10^−3^ (static system) and 2.35 × 10^−4^ cm/min (fluidic system) in the cell-based experiments, and, consequently, were about 2–3.5 times lower compared to the permeabilities for caffeine.

Fluidic permeation studies were performed using the MCmB QuasiVivo^®^ system, that has also been employed in other studies [32,33,34]. Interestingly, in all of the fluidic experiments, the apical concentration measured at time t = 15 min was lower compared to the concentrations measured at t = 75 min and t = 120 min. This observation was assumed to be due to the fluidic experimental setup. After dose administration and additional rinsing with 0.5 mL, drug molecules were pumped from the medium reservoir in the direction of the apical part of the lung chamber and back to the apical dosing/sampling port. As a result, the concentrations at the dosing/sampling port were expected to increase in the first few minutes of the experiments until equilibrium was reached in the apical circuit. This assumption could be supported by in silico model simulations, where the concentrations in C_2_ rapidly increased after dose application, reaching its maximum concentration (C_max_) of ~99.5 µM after ~40 min. Thereafter, the concentrations in the apical circuit continuously decreased due to the transfer of drug molecules into the basolateral compartment.

As described by Sun et al. [43], the implementation of medium volume and drug mass removal might be important in in silico modeling approaches. In the fluidic control experiments, the pairwise observed concentrations at 22 h/24 h and 46 h/48 h showed a distinct stepwise decrease in concentrations even though the concentrations were measured only two hours apart. This stepwise reduction could be due to the decrease in volume during sampling. For example, the 0.5 mL sample at 48 h reduced the total apical volume by 4.2%. Hence, a correction of the volume and mass due to sampling was integrated into the in silico modeling process. Of note, the stepwise decrease was also observed in the fluidic cell experiment for caffeine at 46 h/48 h and for diclofenac at 22 h/24 h, while a stepwise increase was observed for caffeine at 22 h/24 h and for diclofenac at 46 h/48 h.

Some discrepancies between the measured and model-simulated concentrations could be observed. Simulated “steady-state” (t > 24 h) apical and basolateral diclofenac concentrations in the static experiments were slightly lower than the observed concentrations. This may be ascribed to analytical misspecifications, as the average sum of the measured amount of diclofenac in the apical and basolateral compartments at these time points was ~14% (control) and ~10% (cell experiment) higher than the actual amount of substance applied at the beginning of the experiment. Additionally, in the fluidic control experiments, the basolateral concentrations that were observed within the first two hours were slightly underpredicted, while the basolateral concentrations at later time points were predicted precisely.

The residual variability estimated from the static permeation experiments was carried over to the fluidic model without acknowledging potential additional system-dependent variability from the fluidic setup. Comparing fluidic model simulations and the observed concentrations, the predicted variability in the in silico model also covered most of the observed variability in the fluidic scenario, with some exceptions for the apical concentrations and caffeine basolateral concentrations in the control experiments, with some exceptions.

As previously described, the static permeation study was implemented with a bidirectional single-barrier model [11]. While more detailed models that incorporate additional compartments, representing unstirred water layers or cellular compartments, for example, have been used in recent studies [11,13,44], the employed two-compartment approach was a reasonable choice to describe the measured in vitro concentration–time profiles. Here, the single-barrier in silico model was selected, aiming for minimal sufficient model complexity to describe substance permeation. As a result of the employed model, the estimated apparent permeabilities represent composites of all permeation barriers involved, including the apical and basolateral unstirred water layers, the porous membrane, and the lung cell barrier if applicable [11].

In this study, a single-barrier approach was used to model substance permeation. Following this, in a second step, the intracellular pulmonary concentrations could be estimated and integrated in the in silico model. These would be helpful for the mechanistic implementation of active processes for substances that are subject to active transport, intracellular sequestration, or metabolism [10]. Here, potential metabolism of caffeine and diclofenac in the pulmonary epithelial cells seemed to be negligible, as the mean mass balance of the static cellular experiments showed a recovery that was close to the applied dose (39.4 µmol caffeine, 44.2 µmol diclofenac at steady state; applied dose: 40 µmol).

While physicochemical fluxes were integrated via the employed fluidic system, two other major stimuli acting on cells in vivo—biochemical stimuli from other cells (i.e., cytokines, hormones, etc.) and structural stimuli from the three-dimensional (3D) microenvironment [14,15]—could not be considered in this work. Studies have demonstrated more physiologically relevant behavior regarding morphology, cell–substrate interactions, and proliferation rates, among others, in 3D cell cultures [45,46]. In lung cells, differences have been observed regarding the maturation and functionality of the epithelial barrier, with lower TEER and reduced expression of tight junction proteins under 2D conditions [47]. Here, the 16HBE epithelial cell line was used as a proxy to mimic the pulmonary barrier in the lung chamber. Additionally, the study was performed under submerged conditions (apical and basolateral compartment embedded in a medium-filled fluid circuit) and not as an air–liquid interface model. The in vitro system could be adapted in future approaches, integrating, for example, cocultures of multiple cell types in a 3D system to increase physiologic relevance [14,15,48].

Furthermore, the integration of primary cells and additional tissues in multi-organ models could enhance physiologic characteristics in future studies [14,15,49]. Here, a second chamber (C_8_) was already integrated into the in vitro–in silico model of this study, providing the option to integrate additional tissues in the fluidic system. When experimental data from a fluidic system integrating lung cells and additional cell lines (e.g., hepatic cells) become available, the in vitro–in silico framework can be further evaluated for the prediction of these fluidic scenarios. With that, combination of in vitro–in silico models holds the potential to investigate tissue–tissue interactions in multi-organ models and to use modeling and simulation techniques to predict yet untested scenarios, such as drug−drug interactions or pharmacodynamic effects after the hepatic bioactivation of a prodrug molecule [7,25,29,31].

## 4. Materials and Methods

### 4.1. Standard Cell Cultivation

The bronchial cell line 16HBE was cultivated in Dulbecco’s modified Eagle medium (DMEM/F12 with L-glutamine and HEPES; Gibco 11039-021, Thermo Fisher Scientific, Waltham, MA, USA), supplemented with 10% fetal bovine serum (FBS, S0615-500mL, Sigma Aldrich, St. Louis, MO, USA), 100 U/mL penicillin, and 100 µg/mL streptomycin (Gibco 15140-122, Thermo Fisher Scientific). The cells were cultivated in a humidified atmosphere at 37 °C and 5% CO_2_. The bronchial cell line 16HBE was obtained from Dr. Dieter C. Gruenert (Cardiovascular Research Institute, University of California) [50].

### 4.2. Transepithelial Electric Resistance (TEER) Measurements

TEER measurements were performed with a cellZscope+ (Nanoanalytics GmbH, Münster, Germany). The TEER of the in vitro model represents a parameter for the integrity of the cell barrier and the functionality of the tight junctions [51]. Measurements were performed at 37 °C every hour for five days.

### 4.3. Cell Viability Assay

To verify that the fluidic conditions did not negatively affect cell viability, the viability of the 16HBE cells was determined by the alamarBlue^™^ assay (10099022, Invitrogen/Thermo Fisher Scientific) according to the manufacturer’s operating instructions three days after cultivating the cells under fluidic conditions (as described below). The fluorescence intensity was determined at ex/em 560 nm/610 nm. The viability of the cells that had been cultured under static conditions was set to 100% as a reference.

### 4.4. Sample Preparation for In Vitro Permeation Studies

For the in vitro permeation studies, a stock solution of caffeine (Sigma Aldrich #27602-250G) and diclofenac sodium salt (Sigma Aldrich #93484-100MG) was prepared in water (caffeine) and methanol (J.T. Baker, Phillipsburg, NJ, USA) (diclofenac), respectively, with a concentration of 1 mM. Solubilization of caffeine was enhanced using an ultrasonic bath (Elma S15 Elmasonic, Singen, Germany) for 10 min. For each in vitro study, the concentration of the applied solution was measured by HPLC to verify the solubility of the chemicals.

### 4.5. Permeation Studies under Static Conditions

**Figure 7:**
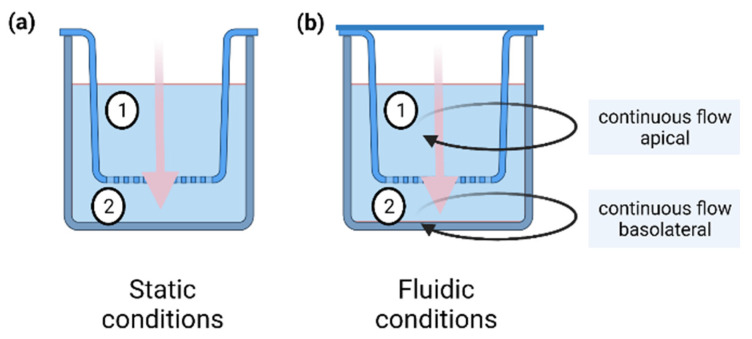
Schematic overview of the static (**a**) and the fluidic (**b**) setup for in vitro permeation studies. Under static conditions an insert was placed in a 24-well plate. The test substance was applied to the apical side (1) and permeated to the basolateral side (2) over time. Under fluidic conditions an insert was placed in a fluidic chamber (QuasiVivo^®^ QV600, Kirkstall) and connected to a fluidic circuit. The test substance was applied to the apical circulation and permeated through the barrier to the basolateral side over time. Black arrows represent the apical (1) and basolateral (2) circuits. Both circuits were connected to a peristaltic pump that ensured a continuous flow of 200 µL/min during the experiment. Pink arrows depict permeation through the barrier.

Static permeation studies were performed in cell culture inserts with a pore size of 0.4 μm and a growth area of 0.6 cm^2^ (PIHP 012 50, 12 mm Diameter Millicell Culture Inserts, Merck Millipore, Burlington, MA, USA) (Figure 7a). For the in vitro cell permeation studies, 4.3 × 10^4^ 16HBE cells were seeded in the cell culture insert. 400 µL cell culture medium (CCM) was added on the apical side, and 600 µL was added on the basolateral side. The cells were cultivated in a 24-well plate (662160, Greiner bio-one, Frickenhausen, Germany) at 37 °C for five days under a physiological pH in the range of 7.0–7.4 with one CCM exchange after two days. Thereafter, CCM was removed, the stock solution was diluted 1:10 with CCM, and the cells were incubated with 100 µM caffeine or diclofenac on the apical side of the insert. The basolateral compartment was filled with fresh CCM. After model substance exposure, total apical, and basolateral CCM were collected each in a 1.5 mL tube (Kisker Biotech GmbH & Co. KG, Steinfurt, Germany) at the following eight time points: 15 min, 1 h 15 min, 2 h, 22 h, 24 h, 46 h, 48 h, and 72 h. For each time point, a separate cell culture insert was used. Samples were then stored at 4 °C until further analysis via HPLC.

In addition to the setup with 16HBE cells, control experiments (without cells) were performed with caffeine and diclofenac (porous membrane as permeation barrier) by following the same procedure described above for the in vitro cell-based study.

### 4.6. Permeation Studies under Fluidic Conditions

**Figure 8:**
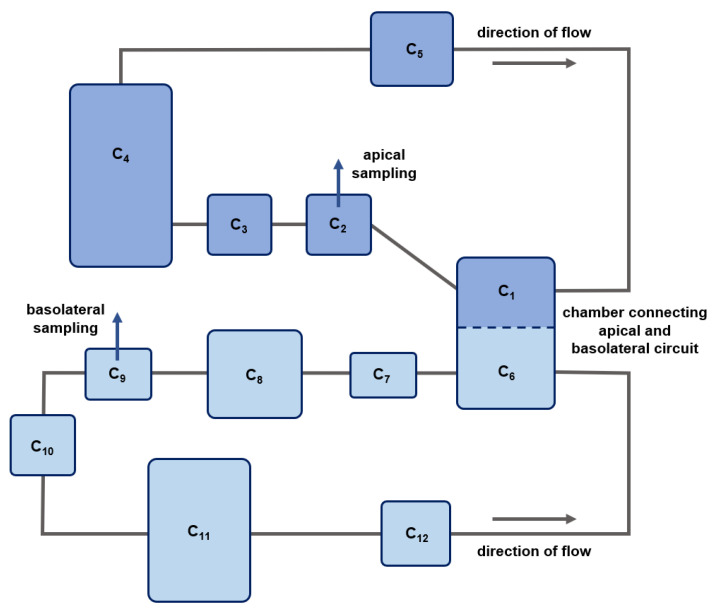
Schematic overview of the in silico model compartments for the fluidic system. Dark blue boxes represent compartments of the apical circuit, and light blue boxes represent compartments of the basolateral circuit. Gray solid lines depict medium flow. The blue dashed line represents the porous membrane without (control) or with the 16HBE cellular barrier. C_1_ and C_6_: apical and basolateral compartments of the lung chamber; C_8_: second chamber; C_2_ and C_9_: apical and basolateral sampling ports including preceding tubes; C_4_ and C_11_: apical and basolateral medium reservoirs; C_3_ C_5_, C_7_, C_10_ and C_12_: connecting tubes.

Fluidic permeation studies were performed in the MCmB QuasiVivo^®^ QV600 (Kirkstall), which was assembled according to the manufacturer’s instructions. An apical and basolateral flow with a flow rate of 200 µL/min was used (Figure 7b). Continuous flow was achieved with a peristaltic pump (ISM597, Ismatec, Cole-Parmer GmbH, Wertheim, Germany). The apical circuit consisted of the apical reservoir bottle, the apical part of the pulmonary chamber, the pump, a three-way cock (B.Braun 4095111; dosing/sampling port; in order of medium flow), and connecting tubes. The basolateral circuit comprised the basolateral reservoir bottle, the basolateral part of the pulmonary chamber, a second chamber allowing the integration of additional cell tissues, the pump, the basolateral sampling port (in order of medium flow), and connecting tubes (Figure 8). Different tubing (Kirkstall QV TS-2 Tubing set; Cole-Parmer GZ06422-01; VWR 228-1066, Radnor, PA, USA) was used to establish the optimal connection between the reservoir bottles, cell chambers, pump, and sampling ports.

For the in vitro cell permeation studies, 4.3 × 10^4^ 16HBE cells were seeded in a cell culture insert with a pore size of 0.4 μm and a growth area of 0.6 cm^2^ (PIHP 012 50, Merck Millipore) and cultivated at 37 °C for five days. Thereafter, the cell culture medium was removed completely, and the insert was positioned in the fluidic QuasiVivo^®^ QV600 (Kirkstall) setup. Both the apical and basolateral circuits were filled with CCM. To adapt the cells in the QV600, the cells were cultured for 30 min with a flow rate of 200 µL/min. For the application of the model substances (caffeine or diclofenac), the flow was stopped, and the stock solution of the substance was injected via the sampling port with a dilution resulting in 100 µM caffeine/diclofenac in the apical circuit. Dosing resulted in both the apical and basolateral circuits being filled with 15 mL CCM. Subsequently, the cells were cultivated for three days under fluidic conditions at a constant flow rate of 200 µL/min. At each of the eight time points (15 min–72 h), samples were collected by opening the sampling port, so that the media could drip from the circuit into a 1.5 mL tube (Kisker). After collecting 500 µL CCM from the apical circuit, the sampling port was closed, and the procedure was repeated for the basolateral circuit.

In addition to the setup with 16HBE cells, control experiments (without cells) were performed with caffeine and diclofenac (porous membrane as permeation barrier), following the same procedure as the one described above for the in vitro cell-based study.

### 4.7. High-Performance Liquid Chromatography (HPLC) Analysis

After the static and fluidic permeation studies, the model substances in the apical and basolateral CCM were quantified via HPLC measurements as described in Elberskirch et al. [52]. The amounts of caffeine and diclofenac were determined by an Agilent 1260 HPLC (Agilent Technologies, Santa Clara, CA, USA). Separation was performed using a Poroshell 120 EC-C18 column (2.1 × 100 mm, 2.7 µm; 695775-902, Agilent Technologies) connected to a guard column (Poroshell 120 EC-C18, 2.1 × 5 mm, 2.7 μm, Agilent Technologies). The mobile phase consisted of three running agents: water (solvent A; sterile filtered 0.2 µm pore), acetonitrile (Solvent B; J.T. Baker), and 0.1% trifluoroacetic acid (TFA) in water (Solvent C; Fisher Scientific). The gradient was programed as presented in Table 2. The flow rate was set to 0.4 mL/min with 5 µL of the sample injected. The column temperature was set to 60 °C (caffeine) and 30 °C (diclofenac), respectively. For detection, a diode array detector (DAD) was used at a wavelength of 275 nm.

### 4.8. Software

For in silico modeling, NONMEM^®^ version 7.4 (Icon Development Solutions, Ellicott City, MD, USA) and the programming language R (version 3.6.3, R Foundation for Statistical Computing, Vienna, Austria [53]) with the *mrgsolve* package version 0.11.1 [54] were used. Further, R was employed for data processing and plot generation.

### 4.9. In Silico Model for the Static System

To model the static in vitro permeation experiments (control and cell experiments), a bidirectional single-barrier model was implemented as a two-compartment ODE system. Since an equilibrium with similar apical and basolateral concentrations was reached for both of the modeled compounds after ~24 h, permeabilities from the apical to the basolateral compartment and vice versa were assumed to be equal. The ODEs describing the changes in concentrations of the model substances in the apical and basolateral compartments over time are depicted in Equations (1) and (2)

(1) $$\frac{dc_{1}\left(t\right)}{dt}=\frac{P_{12,\;i}\times A\times (c_{2}-c_{1})}{V_{1}}\;$$

(2) $$\frac{dc_{2}\left(t\right)}{dt}=\frac{P_{12,\;i}\times A\times (c_{1}-c_{2})}{V_{2}}$$

with the initial conditions $c_{1}\left(0\right)=\frac{D_{s}}{V_{1}}$ and $c_{2}\left(0\right)=0$.

Here, *P*_12,*i*_ represents the permeability of compound *i* (i.e., caffeine or diclofenac) in the control (only porous membrane) and 16HBE cell experiment, respectively. Thereby, *P*_12,*i*_ reflects the compound permeability through all present permeation barriers in the respective experimental setup, such as unstirred water layers, the porous filter membrane, and cell monolayer (apparent permeability) [11]. *A* represents the area of the filter membrane in the lung chamber (here, 0.6 cm^2^), $c_{1}$ represents the drug substance concentration in the apical compartment, and $c_{2}$ represents the concentration in the basolateral compartment. *V*_1_ (0.4 mL) and *V*_2_ (0.6 mL) represent the corresponding volumes of the compartments, while *D_s_* is the dose applied to the apical compartment at time t = 0 (here, 40 nmol).

### 4.10. In Silico Model for the Fluidic System

Figure 8 depicts a schematic overview of the developed in silico model for the fluidic system. Here, C_1_ represents the apical part of the lung chamber and C_6_ the basolateral part. C_2_ comprises the volume of the sampling port and the tubes connecting the lung chamber and the apical sampling port, including the tubes that are clamped in the apical pump. C_3_ represents the tubes connecting the apical sampling port and the apical medium reservoir (C_4_), while C_5_ represents the tubes connecting the apical medium reservoir and the apical part of the lung chamber. The basolateral circuit comprises the basolateral part of the lung chamber (C_6_), a second chamber (C_8_) that provides the option to integrate additional cell tissue cultures in the fluidic system, the basolateral sampling port, including the tubes clamped in the basolateral pump (C_9_), a basolateral medium reservoir (C_11_), as well as connecting tubes (C_7_, C_10_, and C_12_).

In the in silico model for the fluidic system, the compartment volumes were set to the experimentally determined volumes of medium present in the respective compartment at t = 0: 1.5 mL (V_1_), 0.53 mL (V_2_), 0.53 mL (V_3_), 11.62 mL (V_4_), 0.82 mL (V_5_), 2 mL (V_6_), 0.44 mL (V_7_), 2.5 mL (V_8_), 0.49 mL (V_9_), 0.53 mL (V_10_), 8.49 mL (V_11_), and 0.55 mL (V_12_), with V_j_ representing the volume of compartment C_j_. The compartments in the apical circuit and the basolateral circuit, respectively, were connected via medium flows (cf. Figure 8), and the flow rate was fixed to the experimentally determined value of 200 µL/min. The respective ODEs describing the changes in concentration of the model substances in the different compartments over time are depicted in Equations (3)–(5):

(3) $$\frac{dc_{1}\left(t\right)}{dt}=\frac{P_{16,i}\times A\times \left(c_{6}-c_{1}\right)+Q\times \left(c_{5}-c_{1}\right)}{V_{1}}$$

(4) $$\frac{dc_{6}\left(t\right)}{dt}=\frac{P_{16,i}\times A\times (c_{1}-c_{6})+Q\times \left(c_{12}-c_{6}\right)}{V_{6}}$$

(5) $$\frac{dc_{j}\left(t\right)}{dt}=\frac{Q\times \left(c_{j-1}-c_{j}\right)}{V_{j}}\forall \;j\in \left\{2..5,\;7..12\right\}$$

with the initial conditions $c_{4}\left(0\right)=\frac{D_{s}}{V_{4}}$ and $c_{j}\left(0\right)=0\;\forall \;j\in \left\{1..3,\;5..12\right\}$.

Here, *P*_16,*i*_ is the apparent permeability of compound *i* (i.e., caffeine or diclofenac). *A* represents the area of the filter membrane in the lung chamber, which equals 0.6 cm^2^. *c_j_* is the concentration of the drug substance in compartment *j,* and *V_j_* is the corresponding volume of the compartment. Moreover, *Q* represents the flow rate in both the apical and basolateral circuit (200 µL/min), and *D_s_* is the dose of 1.5 µmol applied to the apical circuit at the beginning of the experiments. The dose was applied via a three-way cock followed by 0.5 mL CCM for rinsing. As a result, the applied dose was assumed to be located in the apical reservoir (C_4_) at the beginning of the fluidic experiments.

During sampling, the medium flow was redirected from C_2_ (apical) and C_9_ (basolateral) to a sampling container. Due to continuous flow, the volume of the sampling compartments refilled continuously, allowing the removal of the sample volume. At each sampling time point, 500 µL of medium was extracted from both the apical and basolateral circuits without replacement, reducing their volumes over time. This stepwise decrease in volume in the two reservoirs (V_4_ and V_11_) at each of the sampling time points was incorporated in the in silico model as a zero-order process over the sampling period of 2.5 min.

### 4.11. Model Parameter Estimation and Simulation

During parameter estimation in the static permeation study, permeability parameters (*P*_12,*i*_) were estimated by fitting the model-simulated concentrations to the observed experimental data via first-order conditional estimation method with interaction (FOCE-I) in NONMEM^®^_,_ using a combined proportional and additive residual error model (Equation (6)).

(6) $$Y_{ij}=c_{ij}\times \left(1+\varepsilon_{pij}\right)+\varepsilon_{aij}$$

Here, *Y_ij_* represents the *j*th observed concentration for compound *i* (i.e., caffeine or diclofenac) in the apical or basolateral compartment. $c_{ij}$ is the corresponding model estimated concentration of the drug substance, and $\varepsilon_{pij}$ and $\varepsilon_{aij}$ are the residual errors for the proportional and the additive components of the model with means of zero and variances of $\sigma_{a}^{2}$ and $\sigma_{p}^{2}$, respectively (ε~N [0,$\sigma^{2}$]).

For the stochastic model simulations, the ODEs for the static and fluidic system were implemented in R within the *mrgsolve* framework and used to estimate the 68% prediction intervals (*n* = 500 replicates). Here, the estimated values for the proportional and additive residual errors from the in silico model for the static experiments were carried over to the in silico model for the fluidic system.

For refinement of model predictions in the fluidic cell experiments, a factor was estimated with the model implemented in *mrgsolve* and multiplied with the obtained apparent permeability values from the static in vitro experiments. The factor was estimated via maximum likelihood estimation, assuming a combined proportional and additive error model, minimizing Equation (7) with the L-BFGS-B optimizer from the *nloptr* package [55,56,57].

(7) $$-2LL=\overset{n}{\underset{i=1}{\sum }}\left(\frac{{\left(\hat{y}-y\right)}^{2}}{\left({\hat{y}}^{2}\;\sigma_{p}^{2}+\sigma_{a}^{2}\right)}+log\left({\hat{y}}^{2}\sigma_{p}^{2}+\sigma_{a}^{2}\right)\right)+nlog\left(2\pi \right)$$

Here, y represents the observed concentration, $\hat{y}$ is the corresponding predicted concentration, and *n* is the number of observed concentrations. Further, $\sigma_{a}^{2}$ represents the variance of the additive, and $\sigma_{p}^{2}$ represents the variance of the proportional residual error.

### 4.12. Statistical Analysis

In vitro measurements are presented as arithmetic mean with standard deviation of three independent experiments (*n* = 3) unless mentioned otherwise. Cell viability under fluidic conditions was compared to cell viability under static conditions using an unpaired two-sample t-test performed in Excel (Microsoft Office Professional Plus 2016, Microsoft Corporation, Redmond, WA, USA).

## 5. Conclusions

In summary, this work presents a case example of in silico modeling to describe and predict the concentration–time course of two model substances in a fluidic in vitro permeation system. Here, the estimated apparent permeabilities from static in vitro experiments could be used to predict the behavior of the compounds in the fluidic system under control conditions (with the filter membrane as permeation barrier). In contrast, a decreased permeability in the fluidic setting compared to the static experimental design had to be estimated for a precise description of the observed caffeine and diclofenac concentrations in the fluidic cell experiments with 16HBE cells. The developed models can be further expanded and employed to model the kinetics and the drug effects of pharmacologically active substances in future studies, including experimental scenarios with multiple tissues integrated into the fluidic system. With that, the in vitro–in silico models can contribute to a reduction in preclinical animal studies in line with the 3R principle.

## Figures and Tables

**Table 1:** Parameter estimates for caffeine and diclofenac in the static permeation study.

Parameter	Value	RSE (%)
*Control experiments (without lung cell barrier)*		
P_12, caffeine_ (cm/min)	3.27 × 10^−3^	0.1
P_12, diclofenac_ (cm/min)	2.51 × 10^−3^	0
$\sigma_{a}^{2}$ (µM)	21.4	35
$\sigma_{p}^{2}$	0 (*fixed*)	-
*Cell experiments (with lung cell barrier)*		
P_12, caffeine_ (cm/min)	2.62 × 10^−3^	0.7
P_12, diclofenac_ (cm/min)	1.38 × 10^−3^	0.9
$\sigma_{a}^{2}$ (µM)	2.38	30
$\sigma_{p}^{2}$	0.00596	23

P_12_, apparent permeability from the apical to the basolateral compartment and vice versa; RSE, relative standard error for the fitted parameter; $\sigma_{a}^{2}$, variance of the additive residual error; $\sigma_{p}^{2}$, variance of the proportional residual error.

**Table 2:** HPLC gradient conditions according to Elberskirch et al. [51]. Solvent A: water; solvent B: acetonitrile; solvent C: 0.1% trifluoroacetic acid in water.

Time (min)	Solvent A (%)	Solvent B (%)	Solvent C (%)
0	76.5	8.5	15
0.5	76.5	8.5	15
2.5	42	38	20
4	0	57.5	42.5
10	0	57.5	42.5
14	76.5	8.5	15

## Data Availability

Data is contained within the article.
